# A Rare Case of Pancreatic Divisum Presentation

**DOI:** 10.7759/cureus.37580

**Published:** 2023-04-14

**Authors:** Sam Kara, Saleha Ozair, Miro Brzobohaty, Kester Nedd

**Affiliations:** 1 Department of Neurology, Larkin Community Hospital Palm Springs Campus, Miami, USA; 2 Department of Internal Medicine, Larkin Community Hospital, Miami, USA; 3 Department of Internal Medicine, Larkin Community Hospital Palm Springs Campus, Miami, USA

**Keywords:** magnetic resonance cholangiopancreatography, idiopathic pancreatitis, endoscopic retrograde cholangiopancreatography, recurrent pancreatitis, pancreatic divisum

## Abstract

Pancreatic divisum (PD) is a malformation wherein the majority of affected patients remain asymptomatic or present with complications early in life. Some cases, however, may present in adulthood with recurrent pancreatitis, which makes the diagnosis clinically challenging. Here, we present a rare case of an elderly female with acute-on-chronic epigastric pain secondary to pancreatitis due to PD. During hospitalization, the patient was treated for acute pancreatitis and subsequently discharged with recommendations for corrective surgery. This case is unique particularly due to the older age of onset of symptoms, as well as the lack of exacerbating factors such as drug abuse, alcohol, or obesity. This case highlights the importance of considering PD as a differential diagnosis when managing patients with recurrent pancreatitis regardless of their age.

## Introduction

Pancreatic divisum (PD) is a congenital malformation of the pancreas occurring in less than 10% of the general population, in which the ventral and dorsal pancreatic bodies fail to fuse during early gut formation [1]. As a result of the malformation, most of the pancreatic fluid drains through the accessory papilla [1]. Divisum occurs during the seventh week of fetal development and presents in two distinct forms based on pancreatic ductal drainage, namely, complete and incomplete divisum. Complete divisum accounts for the majority (approximately 70%) of patients, but the likelihood of presenting with symptomatic pancreatitis is equal for both forms [1,2]. While only 5% of divisum patients develop pancreatitis, the biggest challenge is to identify the underlying etiology, especially in patients who have typical risk factors (e.g., obesity, alcohol use, illicit drug use, etc.) and anatomical abnormalities (e.g., narrow major or minor papilla) [2]. Pancreas divisum is typically classified as a type of pancreatic ductal anomaly, which can cause a variety of clinical presentations, including abdominal pain, acute or chronic pancreatitis, and pancreatic ductal hypertension. However, the classification of pancreas divisum can vary depending on the specific clinical context and the underlying etiology of the condition. Normal conservative treatments such as increased fluid intake and a low-fat diet are the first choice for patients with mild acute symptoms [3]. For patients with chronic pancreatitis or multiple episodes of acute-on-chronic exacerbations, invasive treatment is preferred; however, long-term success is variable [2,3]. Options such as sagittal magnetic resonance cholangiopancreatography (MRCP) and surgery to fix papillary obstruction or the anatomical abnormalities present in divisum have seen the most success for long-term relief in patients with recurrent episodes [4,5]. Here, we present the case of a 74-year-old female who presented to the emergency room (ER) with epigastric abdominal pain secondary to an acute-on-chronic episode of pancreatitis.

## Case presentation

**Figure 1:**
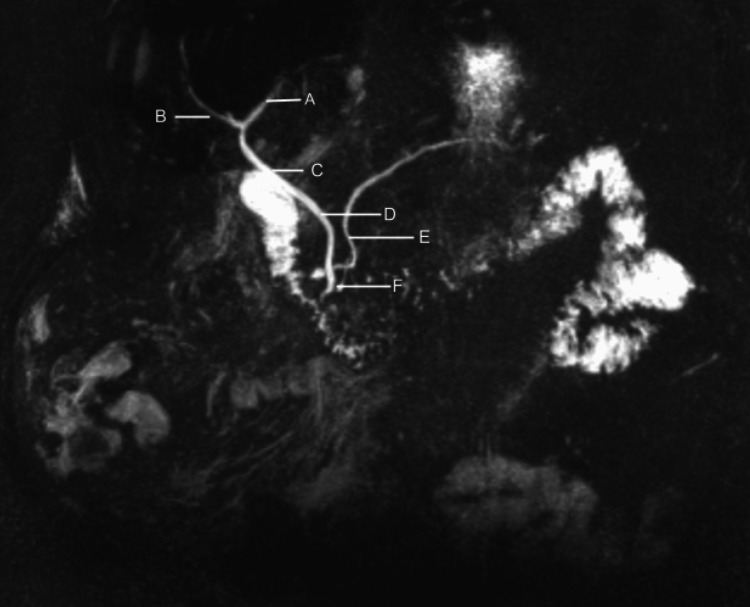
Sagittal magnetic resonance cholangiopancreatography showing pancreatic divisum. A: Left hepatic duct; B: right hepatic duct; C: common hepatic duct; D: common bile duct; E: dorsal pancreatic duct; F: ventral pancreatic duct

A 74-year-old female with a past medical history of chronic pancreatitis, type 2 diabetes mellitus, and gastroesophageal reflux disease presented to the emergency department with upper abdominal pain. The pain was acute in onset; described as constant, dull-aching, non-radiating in nature; 10/10 in intensity; exacerbated by changes in position and with deep palpation; and associated with nausea and vomiting. There were no relieving factors. The patient denied consuming alcohol or tobacco products and reported that she had experienced similar episodes in the past. Her family history was positive for liver cancer in her mother. Vital signs on admission were as follows: blood pressure was 147/78 mmHg, heart rate was 59 beats/minute, temperature was 98°F, and oxygen saturation was 98% on room air. Her body mass index was 30.1 kg/m^2^. Physical examination revealed a soft, nondistended abdomen, with global tenderness on palpation, and normoactive bowel sounds. Her laboratory results showed a normal calcium level, and serum IgG4 was negative; hence, autoimmune pancreatitis was ruled out. Amylase and lipase levels (447 U/L and 10,720 U/L, respectively) were elevated, and a lipid panel was within normal limits. The hematologic profile was within normal limits. Noncontrast computed tomography (CT) of the abdomen revealed acute pancreatitis with a small amount of fluid layering anterior to the left anterior pararenal space, Balthazar score D (image not shown). This was the patient’s third episode of pancreatitis, and as such further investigations were ordered. MRCP done before admission revealed the “crossing-duct” sign, wherein the dorsal pancreatic duct was seen crossing the common bile duct to drain the minor papilla. Consequently, a diagnosis of type 1 PD was made (Figure 1).

During the course of hospitalization, the patient was started on hydration therapy, ondansetron 4 mg orally once a day for nausea, and hydromorphone 0.5 mg IV when needed for pain management. The patient’s symptoms subsequently improved, her pain subsided, and lipase levels trended down to 428 U/L. She was then discharged from the hospital. She was advised not to consume more than 20 g of fat a day, undergo surgical correction per gastrointestinal recommendations, and follow up monthly for six months.

## Discussion

PD is a common congenital pancreatic anomaly with an overall prevalence of about 6% to 10% [3]. Although a vast majority of patients with PD remain asymptomatic, less than 5% are subject to recurrent bouts of seemingly idiopathic pancreatitis [6]. PD is a proposed etiology for both idiopathic recurrent acute pancreatitis (I-RAP) and chronic pancreatitis and is found to be prevalent in about 25-50% of patients diagnosed with I-RAP [3]. The underlying pathophysiology of recurrent pancreatitis in patients with PD involves a transient increase in dorsal ductal pressure during active secretions, which when combined with a narrow papillary orifice leads to inadequate drainage, ductal distention, and, in some cases, acute pancreatitis [3,4].

The symptoms in patients with PD range from asymptomatic to recurrent attacks of mild or severe acute pancreatitis. In symptomatic patients, diagnosis can be confirmed by a secretin-enhanced MRCP, which accurately demonstrates the anomaly [6,7]. In our patient, the MRCP revealed predominant drainage through the dorsal duct, and the classical “crossing-duct” sign specific for the diagnosis of type 1 PD was noted (Figure 1). Thus, PD was determined to be the most likely cause of this patient’s recurrent attacks of pancreatitis.

For mild symptoms, conservative management is usually sufficient [4]. For patients with chronic pancreatitis or multiple episodes of acute-on-chronic exacerbations, endoscopic therapy with sphincterotomy and surgical correction with major papilla sphincteroplasty or stent placement is preferred [4,6]. Furthermore, MRCP was described as a tool in the management of divisum [8]. Failure to recognize PD as a possible etiology of recurrent pancreatitis delays the treatment unnecessarily and leads to additional clinical complications [9]. In the case presented here, the patient had three episodes of pancreatitis before being diagnosed with PD. Initial episodes were managed conservatively and the patient was discharged upon resolution of symptoms without further investigation of the underlying etiology. However, we and others [10] demonstrated that recurrent episodes prompt an MRCP which can accurately demonstrate the defect. This emphasizes the need to have a low threshold for PD, as a differential diagnosis, for recurrent pancreatitis regardless of the age of symptom presentation.

## Conclusions

This case highlights the importance of considering even rare etiologies such as PD in unexplained cases of recurrent pancreatitis. Clinicians should be encouraged not to overlook congenital anomalies as a possible etiology solely based on the age of presenting symptoms. Early confirmation of the underlying etiology and aggravating factors can improve patient outcomes.
